# An unusual lymphoid lesion mimicking meningioma

**DOI:** 10.1111/bpa.12995

**Published:** 2021-07-13

**Authors:** Tammam Abboud, Lidia Stork, Hans‐Ulrich Schildhaus, Christine Stadelmann, Veit Rohde, Dorothee Mielke

**Affiliations:** ^1^ Department of Neurosurgery University Medical Center Göttingen Göttingen Germany; ^2^ Institute of Neuropathology University Medical Center Göttingen Göttingen Germany; ^3^ Institute of Pathology University Medical Center Göttingen Göttingen Germany; ^4^ Institute of Pathology University Medical Center Essen Essen Germany

**Keywords:** lymphoid follicular hyperplasia, lymphoid hyperplasia, lymphoma, meningioma

## Abstract

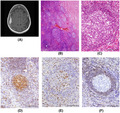

BOX 1 **Slide scan**

Access the whole slide scan at http://image.upmc.edu:8080/NeuroPathology/BPA/BPA‐21‐02‐MISC‐029.svs/view.apml

## 1 CLINICAL HISTORY AND NEUROIMAGING

**FIGURE 1:**
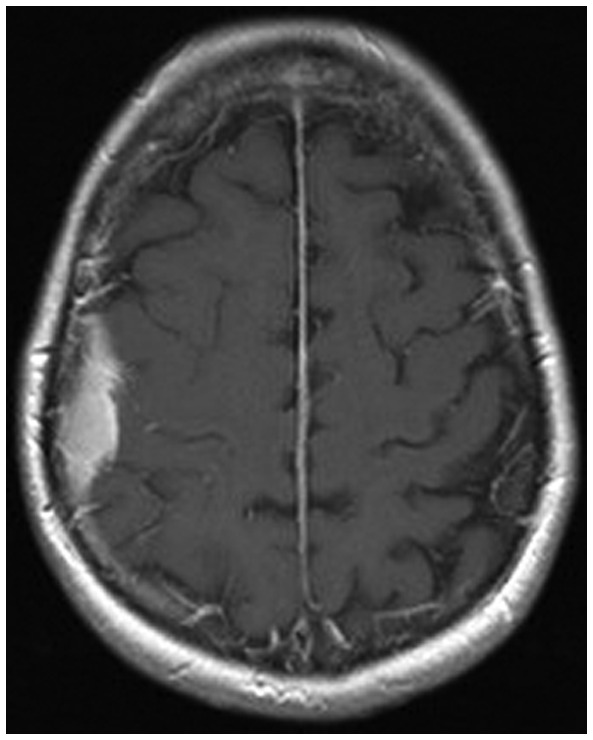
Preoperative MRI (T1 weighted image) showing an en plaque growing mass lesion with homogenous enhancement in the axial plane

A 57‐year‐old woman suffered two epileptic seizures with paresthesia on the left side of the body and dysarthria. Cranial MRI of the patient revealed a contrast medium‐enhancing, en plaque tumor located post centrally over the right parietal lobe (Figure 1). Due to the radiological features of the lesion, we suspected an en plaque meningioma and advised the patient to undergo tumor resection. Microsurgical resection of the tumor was performed via a right‐sided parietal approach. The craniotomy was customized using brain navigation; the tumor was found to be located in the subdural space, adherent to both dura mater and arachnoid, without infiltration of the cerebral cortex. Complete tumor resection was achieved, and the dura above the tumor was resected as well. The extent of resection was graded at Simpson grade I. Postoperatively, the patient had no neurological deficits and was discharged on the seventh postoperative day. Further systemic screening ruled out malignancies and autoimmune disorders. The MRI imaging performed twelve months after surgery showed a complete tumor resection and no signs of recurrence.

## 2 FINDINGS

**FIGURE 2:**
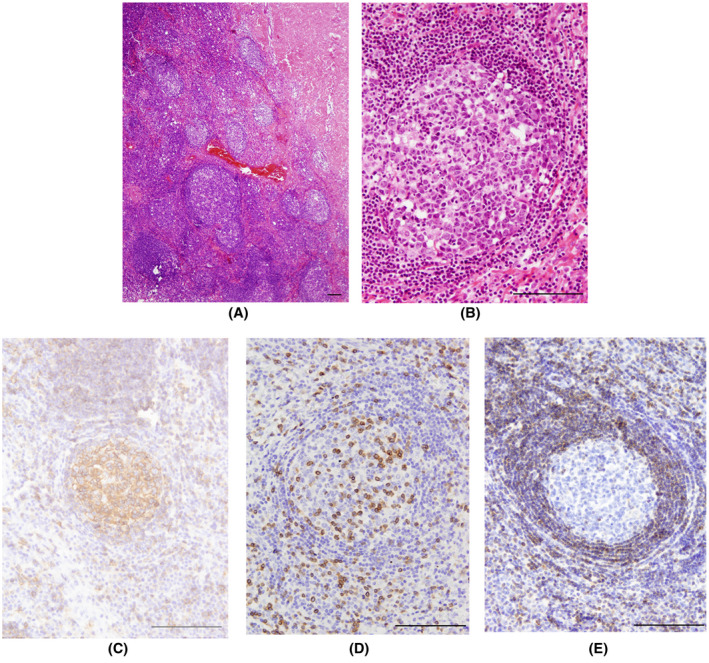
Histopathology of the lesion: Hematoxylin and eosin (HE) staining shows dura matter with multiple lymphoid follicular structures; large interfollicular areas are also seen. The germinal center with polymorphic lymphoid blast cells as well as a sharply demarcated mantle zone with small lymphocytic cells could be clearly distinguished (A). Reactive activated B cells (centroblasts), centocytes, and tingible body macrophages are seen in the germinal center, HE staining (B). Germinal center revealed predominance of the B‐cells, CD20‐Staining (C). CD3‐staining shows multiple T lymphocytes in the mantle zone (D). Bcl‐2 positive B cells are seen exclusively in the mantle zone of the follicle. Scale bar 100 µm (E)

Histopathological examination showed meninges with dense lymphoid follicle‐like structures with a clearly distinguishable reactive germinal center and mantle zone (Figure 2A). A mixed population of blast lymphoid cells was observed in the germinal center, and several tangible body macrophages could be recognized (Figure 2B). Individual mitotic figures were observed. The mantle zone consisted of numerous small lymphocytes and plasma cells (Figure 2B). Cells in the germinal center were positive for CD20 (Figure 2C), CD23, and Pax5 and also showed high proliferative activity (Ki67 was about 90%), where the cells in the mantle zone were positive for CD3 (Figure 2D) and CD8. Immunohistochemical staining for Bcl2 showed positive cells in the mantle zone, but not the germinal center (Figure 2E). In situ hybridization as well as immunohistochemical staining for Epstein–Barr virus was negative. B‐cell clonality studies showed no clonal rearrangement.

## 3 DIAGNOSIS

Reactive lymphoid follicular hyperplasia.

## 4 DISCUSSION

Reactive follicular hyperplasia is a benign reactive proliferation of lymphoid follicles, which may superficially mimic lymphoma. Reactive lymphoid follicular hyperplasia can develop anywhere lymphoid tissue is present. It has been found in the liver, lung, pancreas, thyroid and hard palate, and oral mucosa and has even been described in close proximity to a peripheral nerve. This rare entity is known to be associated with inflammatory and immune reactions and associated with autoimmune diseases such as systemic lupus erythematosus or systemic rheumatic arthritis as well as bacterial and viral infections. A number of cases have been reported in the orbit, in the brain parenchyma, the dura, inside the ventricles and rarely in the pituitary gland under the terms pseudotumor or pseudolymphoma. These reports represent however a heterogenous group of lesions with different morphological features. Only a few bona fide cases of reactive follicular hyperplasia have been described intracranially (1, 2). In these cases, the term pseudolymphoma was used and the lesions showed, as described in our case, involvement of the meninges of the convexity, suggesting a radiological differential diagnosis of meningioma.

Histopathologically, reactive lymphoid follicular hyperplasia should first be differentiated from the follicular lymphoma, but also from other reactive and non‐lymphomatous lymphoid lesions such as Castleman disease, histiocytosis, neurosarcoidosis, polyangiitis nodosa, and granulomatosis with polyangiitis. Some morphological characteristics such as interfollicular areas, which are usually large in reactive follicular hyperplasia, as well as a clearly distinguishable germinal center and the mantle zone may be helpful for differential diagnosis. Polymorphic lymphoid cells and the presence of tingible body macrophages within the germinal center are also indicative of a benign reactive process. The absence of Bcl‐2 positive B cells in the germinal center and lack of clonality of the B cells rules out a diagnosis of follicular lymphoma (3). The absence of S100 positive Langerhans cells, giant cells, necrosis, and an epithelioid cell component is helpful to exclude other differential diagnoses such as histiocytosis, neurosarcoidosis, and particular types of vasculitis. Granulocytes are typically not found in reactive lymphoid follicular hyperplasia. Typical features of Castleman disease, especially vascular proliferation, and hyalinization of the vessel walls are also not seen.

Reactive lymphoid follicular hyperplasia can occur intracranially and mimic a meningioma.

Once resected, no additional treatment is needed. Neurosurgeons and neuropathologists should be aware of this rare entity.

## CONFLICT OF INTEREST

The authors report no conflict of interest concerning the materials or methods used in this study or the findings specified in this paper.

## AUTHOR CONTRIBUTIONS

Tammam Abboud, Veit Rohde, and Dorothee Mielke collected clinical data and drafted the manuscript. Lidia Stork, Christine Stadelmann, and Hans‐Ulrich Schildhaus interpreted histopathology. Tammam Abboud and Lidia Stork prepared the images. All authors read and approved the final manuscript.

## Data Availability

The data that support the findings of this study are available from the corresponding author upon reasonable request.
